# Advanced Plant-Based Glycan Engineering

**DOI:** 10.3389/fbioe.2018.00081

**Published:** 2018-06-14

**Authors:** Laura Montero-Morales, Herta Steinkellner

**Affiliations:** Department of Applied Genetics and Cell Biology, University of Natural Resources and Life Sciences, Vienna, Austria

**Keywords:** plants, glycosylation, *N*- and *O*-linked glycans, glycan engineering, plant-biotechnology, glyco proteins

## Abstract

With respect to biomanufacturing, glycosylation is one of the most addressed post-translational modifications, since it is well-known that the attachment of sugar residues efficiently affects protein homogeneity and functionality. Much effort has been taken into engineering various expression systems to control glycosylation and to generate molecules with targeted sugar profiles. Nevertheless, engineering of *N*- and *O*-linked glycans on well-established expression systems remains challenging. On the one side the glycosylation machinery in mammalian cells is hard to control due to its complexity. Most bacteria, on the other side, completely lack such glycan formations, and in general exhibit fundamental differences in their glycosylation abilities. Beyond that, plants generate complex *N*-glycans typical of higher eukaryotes, but simpler than those produced by mammals. Paradoxically, it seems that the limited glycosylation capacity of plant cells is an advantage for specific glycan manipulations. This review focuses on recent achievements in plant glycan engineering and provides a short outlook on how new developments (in synthetic biology) might have a positive impact.

## Introduction

Plant-based biomanufacturing of (therapeutic) proteins is a relatively new approach with a small number of commercial-scale facilities, although a techno-economic analysis of plant-based platforms estimated a >50% reduction of capital investment and cost of goods compared to mammalian cell expression systems (Nandi et al., 2016). *Solanaceous* species within the genus *Nicotiana*, specifically *Nicotiana tabacum* (tobacco) and *Nicotiana benthamiana* (Australian variety), are currently considered the most appropriate hosts in Molecular Farming. Advantageous features include (i) ease of cultivation and high biomass; (ii) availability of genetic tools for trait manipulation; (iii) amenability to new plant breeding techniques (CRISPR/Cas9); and (iv) non-food status, which minimizes the possibility of contamination of the food supply with industrial designated products.

*N. benthamiana* is particularly well suited for producing recombinant proteins. A significant aspect in *N. benthamiana*'s success is that it contains a natural insertion in the RNA-dependent RNA polymerase 1 gene (Bally et al., 2015), which leads to a reduced level of gene silencing. This feature allows the efficient utilization of transient expression vectors, enabling the rapid production (4–10 days) of high value proteins (hormones, enzymes, antibodies) by transient transfection using agro-infiltration. This success is recently highlighted with the case of ZMapp, an antibody cocktail used during the Ebola outbreak 2014–2015 (Qiu et al., 2014), and the efficient production of vaccines against seasonal flu (D'Aoust et al., 2010). Obtained yields and scalability of the system make it suitable for industrial production. up to 5 g recombinant protein/kg biomass and 10 million doses of a vaccine per months are feasible (Bendandi et al., 2010; Lomonossoff and D'Aoust, 2016). Additionally, *N. tabacum* has been adopted in the past as a biofactory platform by several groups, which have also developed hybrid plants aimed at maximizing productivity (Ling et al., 2012). Despite advantages in using plants as an expression platform major limitations include relatively time consuming transformation protocols associated with moderate protein expression levels when transgenic plants are considered. In addition, the high number of highly active and often unspecific proteases, which are major players of the plant immune system, foster protein degradation and often hinder the expression of functionally active foreign proteins (e.g., Castilho et al., 2014; Niemer et al., 2016).

Notably, plants, as eukaryotic organisms, can be instilled with many of the desirable mammalian traits while maintaining their significant bioprocessing advantages. This facilitates the expression of complex human proteins (e.g., antibodies) with intricate post-translational modifications (e.g., *N*- and *O*-glycosylation). This review concentrates on the ability of plants to produce proteins with targeted glycosylation profiles. A special interest is the generation of targeted human glycoforms at large homogeneity, as this can hardly be achieved by mammalian expression hosts due to their complex endogenous glycosylation machinery. This is of particular interest for the biopharmaceutical industry, since the majority of therapeutic proteins are glycosylated and it is well known that the glycan profiles have a significant impact on protein stability and functionality. In fact, it has been shown that certain protein functionalities can be reversed, depending on the glycosylation profile. Impressive examples are IgG antibodies that exhibit pro or anti-inflammatory activities, depending on their glycosylation profile (Nimmerjahn and Ravetch, 2010).

## Native protein *N*-linked glycosylation repertoire of plants

**Figure 1:**
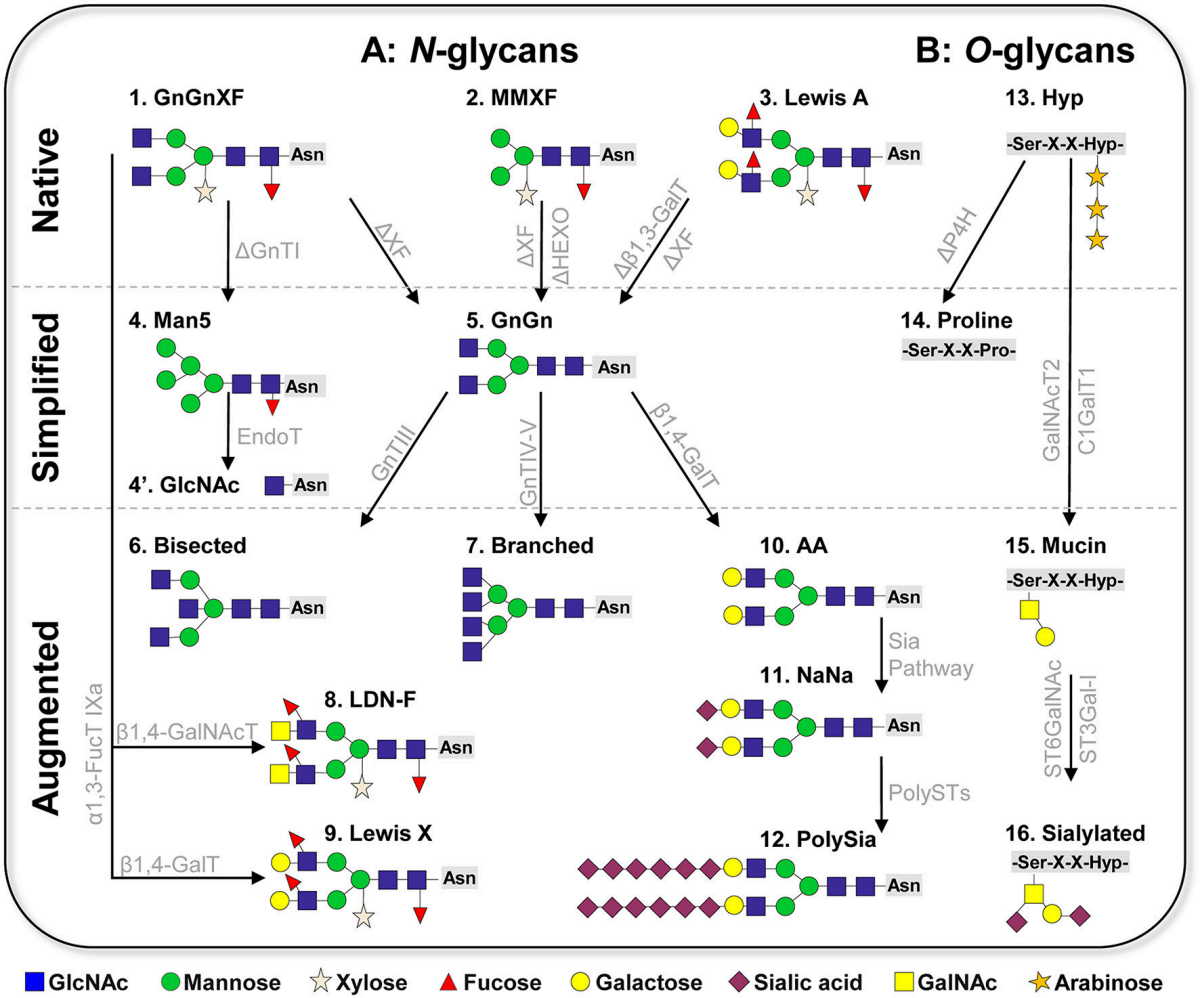
Schematic presentation of plant-based glycan engineering **(A)** *N-*linked glycans. Native: The three major complex glycoforms frequently detected in wild-type plants are 1: GnGnXF, 2: MMXF paucimannosidic structures, and 3: Lewis A structures. Simplified: Glycan simplification and homogeneity by knockout/down approaches (creating the “chassis”). 4: Man5 structure generated by GnTI knockout/down; 4′: single GlcNAc (GlycoDelete); 5: GnGn (common eukaryotic core). Augmentation of plant glycans by stable/transient overexpression of foreign glycosylation enzymes. 6: bisected (GnGnbi) structure; 7: branched ([GnGn][GnGn]) structure; 8: helminth LDN-F structure; 9: Lewis X structure; 10: AA (β1,4-galactosylated structures); 11: NaNa (α2,6-sialylated glycoforms); 12: polysialylated glycans. **(B)** *O*-linked glycans. 13: common native *O*-glycans (hydroxyproline, Hyp, decorated with arabinose residues); 14: PH4 knockout to prevent conversion of prolines to Hyp; 15: generation of mucin type *O*-glycans by the overexpression of human polypeptide GalNAc-transferase 2 (GalNAcT2) and *Drosophila melanogaster* core β1,3-galactosyltransferase (C1GalT1); 16: sialylation of *O*-glycans by overexpression of α2,6- and α2,3-sialyltransferases (ST6GalNAc, ST3Gal-I). ΔXF: knockout/down of α1,3-fucosyltransferase and core β1,2-xylosyltransferase; ΔHEXO: knockout of β-*N*-acetylhexosaminidases HEXO1 and HEXO3; Sia Pathway: recombinant expression of the sialic acid pathway as described by Castilho et al. (2010); PolyST: α2,8 polysialyltransferase II, IV; P4H: prolyl-4-hydroxylase. Glycan nomenclature: Consortium of functional glycomics.

In mammalian cells hundreds of *N*-linked glycoforms can be detected by mass spectrometric analyses on total protein extractions. Such heterogeneity makes a controlled production difficult and hinders batch to batch consistency, an important requirement of the regulatory authorities. Moreover, glycosylation heterogeneity impedes studies to determine the impact of individual glycoforms to a protein's function. Plant cells synthesize only a few *N*-glycan species; in fact, two glycoforms account for >90%. The major one (>60%) are GnGnXF structures, a complex *N*-glycan formation carrying β1,2-linked xylose and α1,3-linked fucose residues attached to the GlcNAc2Man3GlcNAc2 (GnGn) core (Figure 1A, structure 1, nomenclature according to Consortium of functional glycomics). This structure is considered to be the typical plant glycoform and is present in all plant species analyzed (e.g., Wilson et al., 2001). The second major glycoform (20–40%) are paucimannosidic structures, *N*-glycans that lack the two terminal GlcNAc residues at their non-reducing ends (MMXF, Figure 1A, structure 2). This structure, which plants share with many insect cells, is generated in late stages of the *N*-glycosylation pathway due to the terminal trimming of GlcNAc residues by highly specific hydrolases, β-*N*-acetylhexosaminidases (HEXO) (Liebminger et al., 2011). Paucimannosidic *N*-glycans constitute the majority of glycans present on vacuolar glycoproteins and occur in smaller amounts on extracellular plant glycoproteins (Melo et al., 1997; Lerouge et al., 1998). In addition to the two major glycoforms, Lewis A (Le^a^) structures [(FA)(FA)XF] are frequently detected in the plant kingdom (Wilson et al., 2001). They are the only known outer-chain elongation of N-glycans in plants and consist of a trisaccharide formed by β1,3-galactose (Gal) and α1,4-fucose (Fuc) residues to terminal GlcNAc (Fitchette-Laine et al., 1997; Wilson et al., 2001; Strasser et al., 2005; Figure 1A, structure 3).

## Simplification of the plant glycosylation repertoire (generating the chassis)

An important prerequisite for generating homogeneous targeted structures is the establishment of a suitable “chassis,” a minimal structure that acts as an acceptor substrate for further elongation and diversification steps. In terms of complex *N*-glycans, GnGn, the central glycan structure of all higher eukaryotes, acts as such a substrate. To generate a plant-chassis that provides homogeneous GnGn structures, principally the following modifications need to be made: (i) elimination of plant specific residues (i.e., core xylose and fucose), (ii) ablation of HEXO activities, and (iii) elimination of Le^a^ structures. Pioneering work in plant glycoengineering focused on the elimination of the genes that encode β1,2-xylosyl- and core α1,3- fucosyltransferases. Three approaches were successfully applied: genomic mutational knockout, genome editing and gene silencing (sense and antisense RNAi). These resulted in a number of plants and plant cell cultures that indeed synthesize GnGn structures (lacking xylose and fucose residues) at large homogeneity (>90%, Figure 1A, structure 5). So far, seven plant species modified in such a way were reported: *Arabidopsis thaliana* (Strasser et al., 2004), *N. benthamiana* (Strasser et al., 2008; Li et al., 2016), *Nicotiana tabacum* BY2 cells (Yin et al., 2011; Hanania et al., 2017; Mercx et al., 2017), *Physcomitrella patens* (Koprivova et al., 2004), *Lemna minor* (Cox et al., 2006), *Oryza sativa* (Shin et al., 2011), and *Medicago sativa* (Sourrouille et al., 2008), all species that are interesting for recombinant protein production. All glycosylation mutants are viable and do not display any obvious phenotype, at least under laboratory conditions. These plants and cell lines are used to produce different (therapeutic) glycoproteins, including hormones, cytokines and antibodies. The generation of glycoengineered IgG monoclonal antibodies is of particular interest in this context, as it is well-known that the absence of core fucose enhances therapeutic properties such as tumor growth inhibition and viral neutralization (recently reviewed Mimura et al., 2018). Major attention received the production of monoclonal IgG antibodies against EBOLA viruses in glycoengineered *N. benthamiana* (ΔXF) plants, which were used as experimental drug during the last viral outbreak in East Africa 2014–2015 (Qiu et al., 2014; Davey et al., 2016).

The second predominant plant specific *N*-glycoform are paucimannosidic structures (MMXF). Some proteins (endogenous and recombinantly produced) carry exclusively MMXF structures for reasons that are not yet understood (Castilho et al., 2014). Vacuolar glycoproteins are generally considered to carry paucimannosidic structures (Lerouge et al., 1998), however comprehensive vacuolar proteomics/glycomics studies are elusive. Such truncated structures provide an advantage for some therapeutic applications, as shown by carrot cell-produced recombinant human glucocerebrosidase, which is used in enzyme replacement therapies (Shaaltiel et al., 2007). However, when it comes to therapeutic applications, in most cases these structures may negatively affect protein functions, as they can bind to mannose receptors, resulting in rapid clearance from blood circulation (Yang et al., 2015). HEXOs are the target enzymes to prevent GlcNAc hydrolysis from GnGn structures. Gene knockout and silencing approaches were used to eliminate HEXO gene expression, which resulted in the generation of Arabidopsis and *N. benthamiana* mutants with a drastic increase of complex GlcNAc-terminating *N*-glycans at the expense of paucimannosidic structures (Liebminger et al., 2011; Shin et al., 2017). This strategy improves plant glycan homogeneity toward GnGnXF (or GnGn) structures, an important objective in glycan engineering.

Frequently, GnGnXF structures with terminal fucose and galactose (Le^a^ glycans) are detected in various plant species (Wilson et al., 2001; Strasser et al., 2004). Although this glycan formation is barely detected in total protein extracts, some (recombinant) proteins enrich such structures, as shown for recombinant human erythropoietin (EPO) produced in *N. benthamiana* and *P. patens* (Weise et al., 2007; Castilho et al., 2013). Such a glycoform is normally not found in humans, thus it may cause unwanted reactions when used for therapeutic applications. Elimination of Le^a^ epitopes was achieved by a genomic knockout of β1,3-galactosyltransferase gene in the moss *P. patens* (Parsons et al., 2012). This allowed the expression of recombinant EPO with a single *N*-glycan structure, namely GnGn, an obvious improvement when therapeutic application is considered.

Another remarkable approach toward plant glycan simplification and homogeneity is “GlycoDelete” (Meuris et al., 2014) This method relies on the depletion of *N*-acetyltgucosaminyltransferase I (GnTI) activity. GnTI is a key enzyme in the processing of Man5 structures to complex N-glycans, so a GnTI-deficient (cgl) *Arabidopsis* mutant accumulates Man5 structures (Figure 1, structure 4). Subsequent overexpression of the fungal enzyme EndoT, which hydrolyzes high-mannose type glycans (the above-mentioned Man5 structure generated by the cgl mutant), leads to the production of proteins with homogeneous, single-GlcNAc *N*-linked sugars in *Arabidopsis* (Piron et al., 2015, Figure 1A, structure 4′). Such simplified *N*-glycosylation could be used for recombinant proteins in which complex-type *N*-glycans are not required for functionality, e.g., some growth factors, vaccines or even monoclonal antibodies. In addition, these single GlcNAcs are appropriate substrates for further elongation steps, like sialylation, as shown in a mammalian cell glycan engineering approach (Meuris et al., 2014). Notably, GnTI knock down/out lines are valuable sources for the expression of proteins where mannosidic structures are advantageous, as recently demonstrated by the expression of recombinant glucocerebrosidase in *N. benthamiana* mutants (Limkul et al., 2016).

Collectively, these knockout/knockdown approaches are milestones in plant glycoengineering because they (i) demonstrate the ability of plants to tolerate manipulations of the *N*-glycosylation pathway without causing an obvious phenotype, (ii) generate the central acceptor substrate for further elongation/diversification steps, and (iii) increase glycan homogeneity. The latter has substantial implications for downstream processing and meet important regulatory requirements for therapeutic proteins. Notably, there is an ongoing debate on whether plant-specific xylose and fucose residues that are not present in mammals can be tolerated on recombinant therapeutic proteins and whether they present an increased risk for adverse side reactions (Jin et al., 2008; Gomord et al., 2010; Ward et al., 2014; Shaaltiel and Tekoah, 2016; Wang et al., 2017). More importantly, the generation of lines that produce complex N-glycans lacking core fucose and xylose has placed plants in a favorable position for developing next generation drugs, as therapeutic monoclonal antibodies produced in such glycosylation mutants (*N. benthamiana* ΔXF) have improved biological activities (e.g., see above EBOLA virus antibodies).

## Augmentation of plant glycosylation capacities (genomic insertions)

Plant and mammals share a common machinery for the biosynthesis of *N*-glycans that is conserved up to the generation of GnGn structures. With a few exceptions (e.g., Le^a^ structures), plants do not accomplish further diversification of *N*-glycans because they lack the necessary enzymatic catalog. Nevertheless, it seems that plants are equipped with the molecular requirements for mammalian/human-type glycan processing. Approaches to augment plant glycosylation toward human structures are mainly based on the recombinant expression of human glycosylation enzymes (mostly glycosyltransferases, GT) in plants. This is showcased by the generation of bisected (GnGnbi), branched ([GnGn][GnGn]), and galactosylated (AA) *N*-glycans, which are frequently found on human proteins, but are absent in plants (Figure 1A, structures 6, 7, 10). It was found that human GTs maintain their activity when recombinantly expressed in plant cells, i.e., sequentially transferring the respective sugar residues (GlcNAc or galactose) to appropriate acceptor substrates in a highly specific manner (recently reviewed Loos and Steinkellner, 2014). In this context, it was shown that the targeting of GTs to the correct sub-Golgi compartment is of utmost importance. For instance, the recombinant expression of human β1,4-galactosyltransferase interfered with the plant endogenous glycosylation pathway, inducing incompletely processed galactosylated structures (Bakker et al., 2001). In contrast, the overexpression of a fusion construct that targets the enzymes to the *trans*-Golgi compartment enables the synthesis of fully processes di-galactosylated structures (i.e., AA, Figure 1A, structure 10; Strasser et al., 2009). Consequently, human-like glycosylation profiles typical of serum proteins (bisected and branched structures, Figure 1A, structures 6, 7) were engineered in plant-produced glycoproteins by overexpressing modified mammalian GTs targeted to the correct sub-Golgi compartment (Castilho et al., 2011). It is important to note that these structures are generated at large homogeneity on proteins, while serum or mammalian cell produced counterparts usually carry a mixture of several structures. Notably, di-galactosylated structures not only are the major glycoform of important therapeutic proteins (e.g., IgG antibodies), but they also act as acceptor substrates for subsequent sialylation, one of the most complex glycan formation in mammals.

Recent studies demonstrate that even entire human glycosylation biosynthetic pathways can be transfered into plants. This was impressively shown by the introduction of the sialylation pathway in *N. benthamiana* (Figure 1A, structure 11; Castilho et al., 2010, 2012). *In planta* protein sialylation required the overexpression of six foreign genes that need to act at different subcellular compartments in a coordinated manner. The generation of expression systems to synthesize human proteins with a controlled sialylation pattern is of special interest, since these negatively charged sugar residues play essential roles in many aspects of life: e.g., in cell–cell interactions, in cell signaling and in protein stability. A unique feature of sialic acid is that, unlike other sugars, it can form polymeric structures, with its most complex form, polysialic acid (polySia), reaching a degree of polymerization of up to 400 (Sato and Kitajima, 2013). This unique sugar polymer plays multiple roles across different species, from bacteria to humans, e.g., control of immune defense mechanisms, functionality of synaptic contacts, learning and memory (Colley et al., 2014; Hildebrandt and Dityatev, 2015). Thus, the design of such sugar-polymers is of great interest for disease diagnosis and therapeutic applications (like treatment of neurodegenerative diseases). With the overexpression of the human sialic acid pathway together with the human enzymes that catalyze polysialylation (i.e., α-2,8-polysialyltransferases) plant cells were directed toward synthesizing polySia (Figure 1A, structure 12; Kallolimath et al., 2016). Functional assays (e.g., anti-inflammatory activity) point to the high quality of the plant-generated product. This is an amazing achievement, considering the complexity of the structures. The approach relies on a combination of stable and transient expression modules, delivering up to nine foreign genes that need to be expressed simultaneously in a highly synchronized mode in a single plant cell. It demonstrates the enormous flexibility of plant cells for glycan modulation and opens unprecedented ways to study the functional impact of complex glycan structures and to further use them in therapeutic settings.

## Introducing mucin type *O*-glycans

Compared to *N*-glycan engineering, targeted modification of the plant *O*-linked glycans is in its infancies, mainly due to fundamental differences between mammalian and plant *O*-glycosylation pathways. In mammals, the most common *O*-glycan formation on secretory proteins is the attachment of *N*-acetylgalactosamine (GalNAc) to serine or threonine residues (mucin-type *O*-glycosylation). Usually, these GalNAc residues are further modified by the stepwise addition of different monosaccharides (e.g., galactose, GlcNAc, sialic acid), giving rise to diverse mucin-type core *O*-glycan structures that play crucial roles in many different biological processes (Schjoldager and Clausen, 2012). In plants, proline residues are frequently converted to hydroxyproline (Hyp) by prolyl-4-hydroxylases (P4H) which are decorated with arabinose residues (Figure 1B, structure 13), creating a structure not found in mammals. In order to eliminate plant specific *O*-glycosylation, a knockout approach of P4H genes was applied in *P. patens*. The strategy efficiently removed this modification on recombinantly expressed EPO (Parsons et al., 2013), providing the first step in humanizing plant *O*-glycosylation (Figure 1B, structure 14). Importantly, the modification did not cause phenotypical alterations of the production platform. This type of modification was so far applied only to the moss *P. tatens*. Implementation of the mucin-type *O*-glycosylation into higher plants was achieved by overexpression of human polypeptide GalNAc-transferase 2 (GalNAcT2) in *Arabidopsis*, tobacco BY2 cells and *N. benthamiana* (Daskalova et al., 2010; Castilho et al., 2012; Yang et al., 2012; Dicker et al., 2016), which initiated *O*-GalNAc formation on different recombinant glycoproteins (including EPO and IgA1 antibodies). This GalNAc residue serves as a substrate for subsequent elongation with β1,3-galactose by overexpressing β1,3-galactosyltransferase (C1GalT1) (Figure 1B, structure 15). Notably, coexpression of C1GalT1 with the genes for the human sialylation pathway enabled the synthesis of sialylated *O*-glycans (Castilho et al., 2012; Dicker et al., 2016; Figure 1B, structure 16). These studies highlight the ability of engineering plants to produce mammalian-type *O*-glycans, but there are still major shortcomings that need to be addressed. While the conversion of *O*-glycans works efficiently when there is only a single *O*-glycosylation site present (e.g., EPO), only a few sites are modified when several sites exist, as shown for the hinge region of human IgA1 (Dicker et al., 2016). One reason could be the limited supply of nucleotide sugar donors like UDP-GalNAc. Coexpression in *N. benthamiana* of UDP-GlcNAc 4-epimerase (which catalyzes the conversion of UDP-GlcNAc to UDP-GalNAc) and of a UDP-GalNAc transporter yields a more efficient initiation of *O*-glycosylation (Daskalova et al., 2010). Notwithstanding, a major limitation in glycoengineering plant *O*-glycans is the presence of Hyp residues that hinder human-type *O*-glycan modifications. This plant-specific structure may form an immunogenic determinant when present on therapeutic proteins. Thus, efficient knockout strategies to eliminate P4H activities need to be designed.

## Introducing helminth glycosylation into plants

Another interesting aspect in plant glycoengineering is the introduction of complex non-human glycan epitopes. For example, helminth parasites control host-immune responses by secreting immunomodulatory proteins with certain *N*-glycan epitopes (e.g., Lewis X, LDN-F glycan motives). The therapeutic potential of such glycoproteins for the treatment of allergies and autoimmune diseases is anticipated, which increases the interest in their controlled reconstruction. Recent studies exploit plant glycosylation to produce such helminth glycoforms. Overexpression of specific glycosyltransferases (FucTs, GalTs, or GalNAcTs) in *N. benthamiana* enabled the reconstruction of Lewis X and LDN-motives (Figure 1A, structures 8 and 9; Wilbers et al., 2017). Importantly, functional assays revealed immune-stimulatory effects of plant-produced helminth proteins carrying these epitopes, which offers perspectives for therapeutic application and the development of anti-helminthic vaccines.

## Conclusion and outlook

In recent years, substantial progress has been made in glycoengineering plants toward the production of targeted human and non-human structures. Elimination of unwanted native glycosylation enzymes dramatically increases product homogeneity an important feature for product quality and safety. Furthermore, functionality and efficacy of (therapeutic) proteins like antibodies can be specifically modulated and improved. The augmentation of the endogenous glycosylation repertoire by overexpressing foreign glycosylation enzymes (mostly transferases) has led to the production of defined complex *N*- and *O*-glycans, a feat that can hardly be achieved by any other expression system. Custom-designed glycans are valuable tools for identifying biologically relevant glycan structures. Such studies can result in the development of therapeutic molecules with improved activities (a prominent example is EPO) or in the discovery of new glycan-associated functions. A good example of the latter is the discovery of the multiple functions of polySia in immunological and neurological settings (e.g., Hildebrandt and Dityatev, 2015; Karlstetter et al., 2017). This opens new potential medical applications, e.g., the treatment of neurodegenerative diseases (like Alzheimer disease). The interest on such age-related diseases is increasing in view of an aging population. Collectively designed glycan- compounds (free or proteins bound) are fertile ground for the development of products in the era of precision medicine. With the advent of novel powerful methods, like efficient genome editing techniques (CRISPR/Cas9, TALEN), advances in glycoengineering are expected to speed up dramatically, as showcased with the recent generation of plants and cell lines with significantly reduced or totally absent core fucose and xylose (Li et al., 2016; Hanania et al., 2017; Mercx et al., 2017). Genomic modifications that took years to reach completion may now be done within months or weeks using new methodologies. The combination of these techniques with synthetic biology tools (e.g., multi gene assembly, computational biology, bioinformatic tools) enables efficient engineering of even entire (semi-synthetic) biosynthetic pathways (e.g., Kallolimath et al., 2016), and will probably result in unprecedented new insights into glycobiology and their subsequent application in various medical settings in the near future. Overall, plant and non-plant glycoengineering provides strategies to optimize the safety, functionality, and efficacy of therapeutic compounds as more affordable treatment options in the next decade.

## Author contributions

Both authors listed have made a substantial, direct and intellectual contribution to the work, and approved it for publication.

### Conflict of interest statement

The authors declare that the research was conducted in the absence of any commercial or financial relationships that could be construed as a potential conflict of interest.
